# Reporter Cell Assessment of TLR4-Induced NF-κB Responses to Cell-Free Hemoglobin and the Influence of Biliverdin

**DOI:** 10.3390/biomedicines7020041

**Published:** 2019-06-03

**Authors:** Jill Sharma, Taylor Boyd, Claudia Alvarado, Edwin Gunn, Jaimie Adams, Traci Ness, Robert Dunwoody, John Lamb, Brittany House, James Knapp, Ronald Garner

**Affiliations:** 1 Department of Biomedical Sciences, Mercer University School of Medicine, Savannah, GA 31404, USA; jill.sharma@unlv.edu (J.S.); teboyd729@gmail.com (T.B.); Claudia.Y.Alvarado@live.mercer.edu (C.A.); Edwin.Gunn@atriumhealth.org (E.G.); jaimemichele.adams@gmail.com (J.A.); Robert.Perry.Dunwoody@live.mercer.edu (R.D.); John.Morgan.Lamb@live.mercer.edu (J.L.); house.brit@gmail.com (B.H.); knapp_je@mercer.edu (J.K.); 2 Department of Biology, Georgia Southern University, Savannah, GA 31419, USA; tness@georgiasouthern.edu

**Keywords:** NF-kB, hemoglobin, toll-like receptor 4, cancer, reporter cells, immune regulation

## Abstract

Hemoglobin (Hb) released during red blood cell lysis can initiate TLR4-dependent signaling and trigger NF-κB activation in surrounding cells. Observations of chronic bleeding in various cancers leads us to hypothesize that Hb and Hb degradation products released from lysed RBC near cancer nests might modulate local TLR4-positive cells. We addressed the hypothesis in vitro by measuring Hb- and biliverdin (Bv)-induced NF-κB signaling in an engineered human TLR4 reporter cell model (HEK-Blue^TM^ hTLR4). Therein, TLR4 stimulation was assessed by measuring NF-κB-dependent secreted alkaline phosphatase (SEAP). hTLR4 reporter cells incubated with 8 ηM lipopolysaccharide (LPS) or 20-40 μM fungal mannoprotein (FM) produced significant amounts of SEAP. hTLR4 reporter cells also produced SEAP in response to human, but not porcine or bovine, Hb. HEK-Blue Null2^TM^ reporter cells lacking TLR4 did not respond to LPS, FM, or Hb. Bv was non-stimulatory in reporter cells. When Bv was added to Hb-stimulated reporter cells, SEAP production was reduced by 95%, but when Bv was applied during LPS and FM stimulation, SEAP production was reduced by 33% and 27%, respectively. In conclusion, Hb initiated NF-κB signaling that was dependent upon TLR4 expression and that Bv can act as a TLR4 antagonist. Moreover, this study suggests that hemorrhage and extravascular hemolysis could provide competitive Hb and Bv signaling to nearby cells expressing TLR4, and that this process could modulate NF-κB signaling in TLR4-positive cancer cells and cancer-infiltrating leukocytes.

## 1. Introduction

The spontaneous onset of bleeding among cancer patients can occur in a spectrum of presentations, ranging from chronic occult bleeding to sudden profound bleeding [1,2]. Hemorrhages in and around tumor masses are quite common in lung, bladder, gastric, and colorectal cancers where it is attributable to tumor invasion or occlusion of blood vessels. Studies examining hemoglobin (Hb)-induced changes in cancer survival and progression are uncommon in the literature; nonetheless, a novel study by Yin et al. [3] provided new evidence that red blood cells (RBCs) and Hb activate the reactive oxygen species-NF-κB pathway in both tumor cells and macrophages. A more robust collection of surgery studies are available wherein they have examined coronary bypass (Viz., concomitant to 15% intravascular hemolysis), thereby providing a wealthy source of data describing the systemic effects of Hb on the immune response [4,5,6,7,8]. In these data, it is suggested that Hb-induced interleukin-10 (IL-10) may play a role in the termination of inflammatory responses, in part, by promoting regulatory T cell (Treg) differentiation. The authors suggest that this action is accomplished through interleukin-10, -19, and transcription factor Foxp3 expression, which all represent important steps in Th0 to Treg cell differentiation. Bleeding, RBC lysis, and immune regulation have also been examined by Yeh et al. [8], where they show a significant increase in interleukin-19, TNF-α, and CRP. Collectively, these studies show that derivatives from RBC lysis can produce immunomodulation and offer some insight into how hemorrhage might dysregulate immune mechanisms aimed at targeting cancerous cells.

Toll-like receptors (TLRs) are a type of pattern recognition receptor (PRR). These receptors categorically recognize endogenous and exogenous pattern-defined ligands, and their activation contributes to inflammatory responses. Insomuch as chronic inflammation is often linked to cancers, persistently encountered compounds that activate TLRs are implicated as cancer promoters [9,10,11]. For example, TLR4 is a well-recognized PRR that detects pathogen-associated molecular patterns (PAMPs), as well as damage-associated molecular patterns (DAMPs) [12]. Ligand activation of this receptor triggers cytosolic adaptor proteins that induce downstream signaling cascades, which in turn promote the transcription of inflammatory cytokines and enzymes through the release of NF-κB [13]. Moreover, TLR4 responses commonly orchestrate the transcription of inflammatory cytokines, such as TNF, IL-1, and IL-8. Lipopolysaccharide (LPS) is the best-known TLR4 ligand [14], and its activation signal is achieved through both MyD88-dependent and TRIF-dependent pathways, either of which can initiate NF-κB transcriptional signals to the nucleus [15,16]. A host of endogenous ligands—including heat shock proteins, urate crystals, surfactant A, and others [17]—are capable of activating NF-κB through TLR4. Once in the nucleus, NF-κB can initiate transcription of cytokines, activate cyclins, and/or regulate apoptosis.

TLR4-initiated inflammatory responses are seen following hepatic ischemia [18], cardiac ischemia [19], brain hemorrhage [20], and pancreatitis [21]. Prolonged exposure to DAMPs most likely contributes to the mechanisms of inflammation that occur following vascular bleeding. This can be demonstrated through characterization of DAMPs that predominate during and after injury and surgical procedures [19]. In this context, the discovery that Hb activates cells via TLR4, or a TLR4-like mechanism, suggests that accumulation of Hb and Hb-catabolic products could modulate immune responses [22,23,24]. Saturation of Hb-scavenging, haptoglobin-sequestration, and Hb catabolism are potential points for dysregulation of inherent Hb controls [25]. For example, recent studies suggest that biliverdin (Bv) binding to aryl hydrocarbon receptor (AhR) can modulate NF-κB activation by inducing antagonistic gene transcription [26,27,28]. In addition, Bv-induced endothelial nitric oxide (eNOS)-dependent nitrosylation of Bv-reductase (Bvr) causes a conformational change in Bvr that initiates its translocation into the nucleus, where it represses AP-1-dependent TLR4 expression [23]. In view of these findings, we tested the following hypothesis: Bv will modify TLR4-induced NF-κB responses to not only Hb, but also fungal mannoprotein (FM) and LPS. To assess the potential role of TLR4-Hb recognition as a point of immune regulation, we chose to use a commercially engineered TLR4 reporter cell system (Human Embryonic Kidney 293; InvivoGen; HEK-Blue^TM^ hTLR4). This approach focused our study on receptor/ligand compatibility and NF-κB activation. In our lab [29] and others [30,31], studies using a collection of TLR4 ligands have correlated NF-κB dependent secreted alkaline phosphatase (SEAP) production with cytokine transcription. They also demonstrate this model’s utility as reliable, reproducible, and consistent when compared to other TLR4 stimulation models.

TLR4 expression and activation has been shown to correlate with cancer-associated immune dysfunction, enhanced cancer cell survival, cancer progression, and tissue invasion [32,33,34,35]. In the present study, we examined the interaction of Hb and Bv as competitive DAMPs that together trigger TLR4 signaling. In this respect, we evaluated their potential roles in localized anti-cancer immunity, or how they might serve as potential survival signals for cancer cells. In addition to demonstrating that human Hb is a capable TLR4 agonist, we also compared TLR4 activation with bovine, porcine, and human Hb. Our results show that human Hb stimulates NF-κB signaling in HEK-Blue hTLR4 cells, but to a far lesser extent than LPS-stimulation. We also found that bovine and porcine Hb did not stimulate these TLR4 reporter cells. Polymyxin B de-pyrogenation of Hb and Bv had little or no effect on Hb’s ability to stimulate or inhibit reporter cell production of SEAP. We also found that Bv was an inefficient stimulator of the TLR4 response, but that Bv significantly inhibited Hb’s ability to activate cells via TLR4. Inhibition of TLR4-dependent NF-κB activation was also observed when either LPS or FM was used to stimulate reporter cells. Moreover, these data show that with the catabolism of Hb->Bv, Bv can act as an auto-regulatory factor with the potential to reduce Hb’s activation of the TLR4 pathway, but may also impact activation by other ligands.

## 2. Materials and Methods

### 2.1. TLR4 Ligands

FM was extracted from *Candida albicans* 20A that was grown at 37 °C to stationary phase in Sabouraud’s dextrose broth Sigma-Aldrich, St. Louis, MO, USA (Sigma). FM was isolated from fungal cells by the hot citrate method and purified by cetyltrimethylammonium (CTAB) precipitation, followed by alternating steps of bromide and acetylation treatments [29]. FM was stored as a lyophilized powder at 4 °C until needed. *Salmonella* LPS was used throughout and was purchased from Sigma Chemical Company (Sigma). Hb and Bv were also purchased from Sigma. FM was prepared with reagents that were purchased as endotoxin-free (Sigma). All ligands, other than FM, were purchased as endotoxin-free reagents. Prior to their application, ligands were routinely filtered through polymyxin B resin to remove potential endotoxin contamination, as discussed here and elsewhere [29].

### 2.2. Cell Culture and TLR4 Activation Assays

HEK-Blue^TM^ hTLR4 and HEK-Blue^TM^ Null2^TM^ cells were purchased from InvivoGen, San Diego, CA, USA. The cells were cultured at 37 °C in 5% CO_2_ in 25 or 75 cm^2^ vented flasks using Dulbecco’s minimal essential media (DMEM) containing glutamine, heat-inactivated fetal bovine serum (FBS), penicillin/streptomycin, and normocin (InvivoGen). All culture media and plastics were purchased as endotoxin-free, and all glassware was de-pyrogenated by baking at 250 °C for 2 h. Selection of the plasmids in HEK-Blue hTLR4 cells required the use of HEK-Blue^TM^ (InvivoGen), and in HEK-Blue Null2 cells required the use of zeocin (InvivoGen). The cells were harvested for stimulation as non-confluent (50–70% of confluence) using Ca^+^- and Mg^+^-free Hank’s balanced salt solution (HBSS; Sigma). Cells were not centrifuged, as sufficient numbers were available for subculture in the dislodged cells. Clumps were eliminated by sedimentation at 100× *g*. Cell counts and viabilities were determined by hemocytometer and Trypan Blue exclusion. The cells were plated at 0.5–2.0 × 10^5^ viable cells per 100 μL per well on flat bottom 96-well plates (CellStar, Greiner Bio-One, Monroe, NC, USA).

### 2.3. Stimulation and QuantiBlue Assay

Cells were grown to 50–70% confluence (Viz., greater than 70% confluence results in high NF-κB background values) and were plated at a cell density of 0.5–2 × 10^5^ viable cells per well in a 96-well flat-bottomed tissue culture plate prior to treatment. Optimization experiments demonstrated that 1–2 × 10^5^ cells/well. Cells were added to the 96-well plates in 100 μL of compete media. Dilutions were made in separate plates using complete media as the diluent. LPS, FM, Hb, or Bv dilutions were added with a final media volume of 200 µL/well. Relative NF-κB activity was determined by measuring the SEAP activity that accumulated the culture media following overnight incubations with the agonists. The next day, 20 µL of supernatants were removed from each well and transferred to a new 96-well plate containing 180 µL pre-warmed (37 °C), filtered (0.22 µm) QUANTI-Blue^TM^ (a chromogenic substrate for SEAP: InvivoGen). Reactions were developed at 37 °C (without CO_2_) for 10–60 min, SEAP activity was measured at 630 nm using a BioTek Epoch microplate spectrophotometer (BioTek Instruments, Inc., Highland Park, Winooski, VT, USA), and the results were analyzed with Gen5 Data Analysis software (BioTek). Three wells in each plate were read as blanks and the reaction background was subtracted from test OD 630 nm. As noted previously by our lab [29], HEK-Blue Null2 cells serve as negative controls for TLR4 activation as these are non-responsive to TLR4 ligands: SEAP production is induced in the HEK-Blue Null2 control cells by treating them with 100 ng/mL TNFα.

### 2.4. Statistics

All tests and controls were done in matching triplicate or quadruplicate wells, and the average means +/− standard error of the means (SEM) were calculated. The results shown here are representative responses from multiple experiments. When only 2 means were directly compared, Student’s *t*-test was used to determine the significant differences in the means. *t*-test *p*-values <0.05 were required to assign significance and are represented as an asterisk (*) in the figures. Where multiple regression lines were compared, dilution and kinetic curves were drawn to represent a range of concentrations or time dependent activities (e.g., X_1_ and X_2_), and their respective *Y* values were evaluated. The adjusted R^2^ values and slopes of the data lines were calculated and statistically evaluated using linear regression analysis. These data are found in their respective figure legends. Where different TLR4 ligands were compared in the same experiments, the data and regression lines are shown in the same figure. To assess significance between two linear regressions, a factor analysis of variance (ANOVA) was performed on the data groups [36]. Throughout the manuscript, significance is noted as an asterisk (*) in the figures and as *p* < 0.05 in the text.

## 3. Results

### 3.1. TLR4 Stimulation with Hb

**Figure 1:**
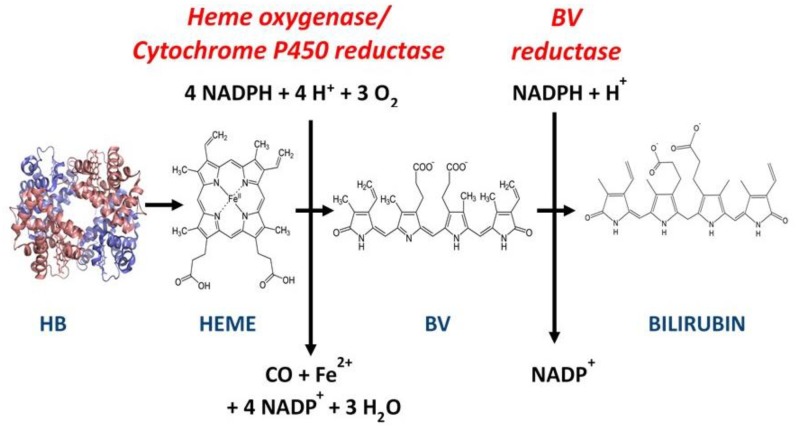
Red blood cells are degraded into hemoglobin (Hb) during cell turnover or damage. Hb further catabolizes to globin chains, which are broken into amino acids and heme. Heme oxygenase catabolizes the heme to yield ferrous iron, which will later travel to bone marrow to be used in new heme production, carbon monoxide, and biliverdin (Bv). Bv is then reduced to an unconjugated form of bilirubin (Bu). All of these degraded components can be present in inflammatory responses related to hemolysis, and each can maintain a unique contribution to the inflammatory reaction. The balanced reaction above is based on Hb with its heme iron in the ferrous state. The molecular image of Hb was generated using PyMol (Version 2, Schrodinger, LLC, Delano Scientific, San Carlos, CA, USA). Similar images of Heme, Bv, and Bu are available through ChemSpider online search and share chemistry, Royal Society of Chemistry, Cambridge, UK.

When released from RBCs, Hb is scavenged for its iron content and also to protect against oxidation [37]. This is achieved, in part, when Hb is degraded to Bv and then to bilirubin (Bu) by the sequential catabolic actions of heme-oxygenase (HO) and Bv reductase (Figure 1). The molecular image shown in Figure 1 is predicted from the PyMol molecular graphics system (PyMol Version 2, Schrodinger, LLC; [38]). Moreover, RBC-derived Bv and Bu can remain in injured tissues until Hb and its catabolites are scavenged or further degraded. During this time, Hb or its catabolites can also signal the immune system and local TLR4-expressing cells via their TLRs. While it has been proposed that both Bv and Bu can inhibit expression of TLR4 [23,39], this observation was made in macrophages and was strictly dependent on endothelial nitric oxide synthase (eNOS) activity. In contrast, HEK293 cell lines do not express eNOS [40], but may express low levels of another NOS isoform. In the present study, we provide evidence that demonstrates Bv’s ability to inhibit Hb’s activation of the TLR4/NF-κB pathway in an HEK293-derived cell line. Bv significantly inhibited LPS and FM activation of the same TLR4/NF-κB pathway, but to an obviously lesser extent than the inhibition seen with Hb activation. We used HEK-Blue hTLR4 cells (InvivoGen) as NF-κB reporter cells throughout these experiments, except where the parental HEK-Blue Null2 cells were used to demonstrate dependency on TLR4 expression. The HEK-Blue hTLR4 reporter cells produce and release SEAP into the media as an NF-κB-dependent action that is dose dependent, with respect to the addition of each TLR4 ligand.

**Figure 2:**
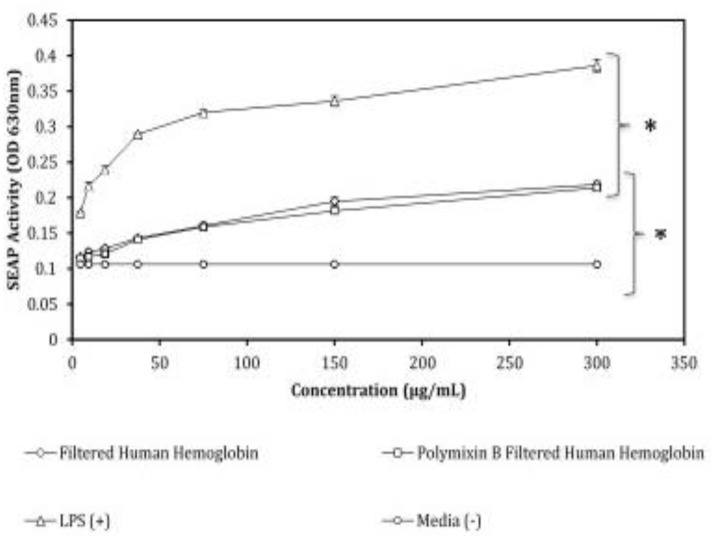
Hb-stimulated HEK-Blue hTLR4 cells in a dose-dependent manner. Total protein in each Hb preparation was assessed by 280 nm absorbance and standardized before application to the cells. Hb (Sigma) was first centrifuged, filtered (0.22 μm), incubated on polymyxin B spin columns, and then incubated with HEK-Blue hTLR4 cells overnight (squares). Twenty μL of media was removed from each well and mixed with the SEAP substrate. The reaction progressed for 30 min and the absorbance at 630 nm was assessed. Data shown is the mean of triplicate samples, and each point includes the standard error of the mean (SEM). Error bars that are not visible are present but fall within the range of the symbol. Standard linear regression analysis was performed, significant differences between regressions were evaluated by ANOVA, and significance has been designated here by brackets and asterisks (*) where *p* < 0.05. HEK-Blue hTLR4 cells treated with (protein) adjusted aliquots of ligands, including Hb saved before polymyxin extraction (diamonds; slope = 0.00031; R^2^ = 0.890), polymyxin treated Hb (squares; slope = 0.0003; R^2^ = 0.89), LPS positive controls (triangles; slope = 0.00044; R^2^ = 0.846), and media alone (circles; slope = 0.00030; R^2^ = 0.90).

**Figure 3:**
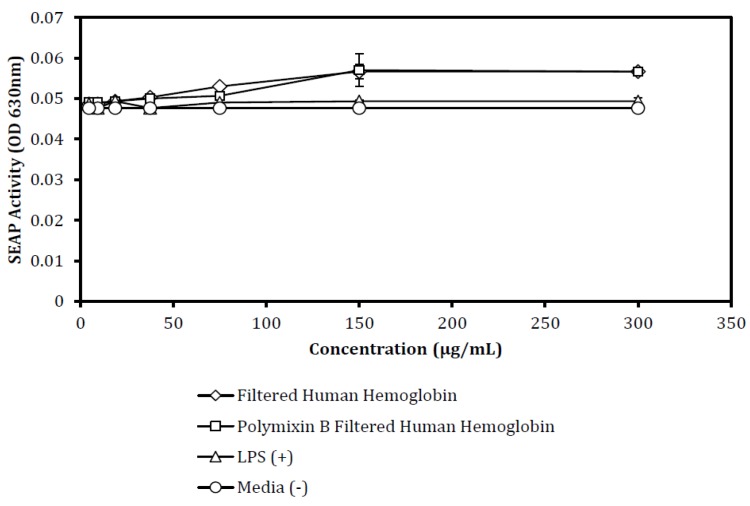
HEK-Blue Null cells that lack the TLR4 expression vector, but express the NF-κB reporter plasmid, were incubated overnight with TLR4 ligands. A serial dilution of each ligand was performed. The non-filtered Hb (Diamonds), polymyxin B extracted Hb (Squares), LPS (Triangles), and control media (Circles) were all prepared as noted in Figure 1. Secreted alkaline phosphatase (SEAP) activity was assessed by removing 20 uL, mixing the aliquot with substrate, and measuring the absorbance at 630 nm. The data is shown as the mean (+/− SEM) absorbance for SEAP substrate conversion. Due to the scaling of the figure, the lines may appear unrelated—however SEAP activity was minimal (0.2% compared to Figure 1 lipopolysaccharide (LPS) control; HEK TLR4 cells) and using ANOVA comparisons of the regressions, no significant difference was observed between polymyxin B-treated and non-treated HEK-Blue Null2 (*p* > 0.05).

Activation of NF-κB occurred in response to Hb treatment (Figure 2). In the preparation of Hb, we found that removing aggregates from reconstituted lyophilized Hb by centrifugation at 12,000× *g* was essential to achieve optimal Hb stimulation. The reconstituted, centrifuged, and sterile-filtered Hb (0.22 μm) was next incubated on polymyxin B spin columns (Sigma) for 1 h to remove any or all LPS contamination. For comparison, some Hb aliquots were taken after filtration, but before LPS depletion, and these were then diluted to the same protein concentration as the Hb filtered through polymyxin B spin-columns (Sigma). Serial dilutions of both Hb preparations were applied to the HEK-Blue hTLR4 cells, as were serial dilutions of LPS (positive control) and culture media (negative control). Each ligand-stimulation was assessed in quadruplicate wells in a 96-well plate. The concentrations tested ranged from 5 ug/mL through 300 ug/mL for each ligand. The SEAP activity in each stimulated culture was measured as the OD 630 nm of the enzyme’s product, which accumulated from Quanti-Blue^TM^ (InvivoGen) conversion. As expected, LPS stimulation of the cells yielded robust SEAP activities that were representative of strong NF-κB signals [29]. Evaluation of the linear portion of the stimulation curves (75–300 μg/mL) suggested that Hb stimulation of NF-κB-dependent SEAP activity was roughly 57% (*p* < 0.05) of that observed with LPS stimulation when 150 μg/mL Hb was used. Hb-induced SEAP activity was roughly equivalent between the two ligands when 300 μg/mL Hb (55% of LPS response) and 75 μg/mL Hb (50% of LPS response) were used. By comparing the two dilution curves, it was clear that filtration through polymyxin B had little or no effect (<7% decrease with 150 μg/mL Hb) on Hb’s ability to stimulate HEK-Blue hTLR4 cells. No polymyxin-dependent change in Hb-inducible SEAP activity was observed at 75 μg/mL or 300 ug/mL Hb. Moreover, these data suggest that Hb’s ability to stimulate SEAP was significantly inferior to that of LPS (*p* < 0.05), and that based upon polymyxin B-depletion, Hb stimulation was not caused by LPS contamination. LPS, Hb, and polymyxin filtered-Hb were tested in the parental HEK-Blue Null2 cells. These cells possess the NF-κB-dependent SEAP plasmid, but lack TLR4. As is shown in Figure 3, none of the ligand preparations stimulated these control cells, suggesting that Hb stimulation in this cell model was TLR4-dependent. This HEK-Blue Null cell line responded with SEAP production when stimulated with TNF [29], an action that is not TLR4-dependent.

**Figure 4:**
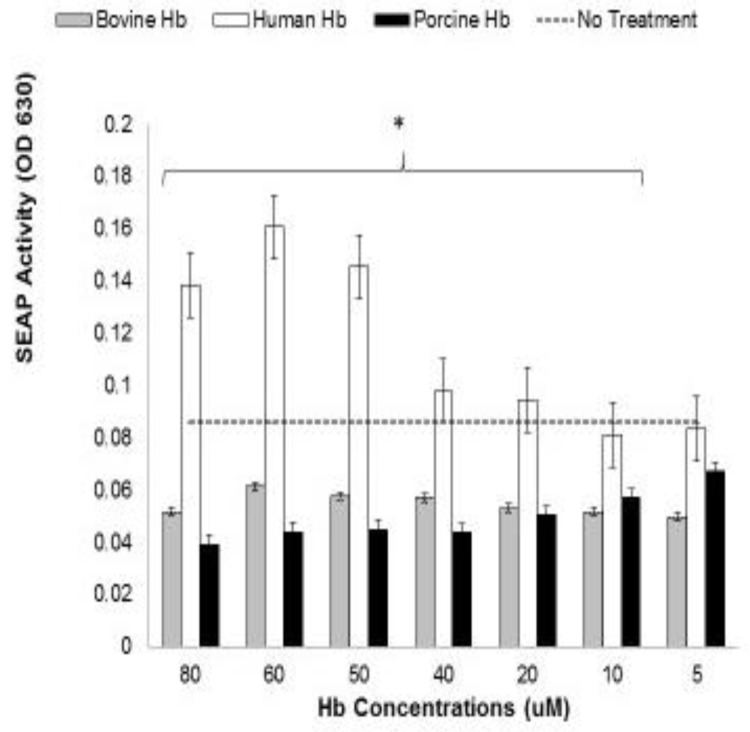
Comparison of TLR4 stimulation and NF-κB demonstrated by Hbs derived from different species (xenogeneic Hb). Human, bovine, and porcine Hb (Sigma) were diluted to equal molar concentrations before addition to HEK hTLR4 cells. After 24 h incubation with the ligands, 20 µL supernatant aliquots were added to QUANTI-Blue^TM^ substrate solutions and incubated for 20 min. The OD 630 nm was measured. Data shown here are the means of quadruplicate treatments (+/− SEM) and the data are drawn as multiple bar graphs to compare the independent SEAP values respective to the background SEAP production (dashed line) with HEKTLR4. ANOVA comparisons between the human Hb and each of the other Hbs are designated by a horizontal bracket, and the asterisk (*) denotes significance over that entire range of human Hb dilutions where *p* < 0.05.

The potential complication of xenogeneic Hb reactivity with TLR4 exists within the cellular hTLR4 reporter system used here, Viz.; this system relies on xenogeneic bovine serum for cultivation. For example, had calf serum stimulated a high background level of TLR4 signaling, a serum-free culture media might have been required for HEK-Blue hTLR4 reporter cell testing of Hb. Furthermore, it was important to learn if dietary or therapeutic xenogeneic Hbs might also have the potential to stimulate TLR4-expressing cells. Xenogeneic Hb preparations from two other species were compared as TLR4 ligands using HEK-Blue hTLR4. All Hbs were compared at equal molar concentrations. Serial dilutions were made on each Hb to account for characteristics that might be revealed by increased or decreased dosage. SEAP conversion of the QuantiBlue (InvivoGen) substrate was measured for 40 min the day after Hb stimulation. The data in Figure 4 show that, of the three Hbs tested (human, bovine, and porcine), only human Hb acted as a TLR4 agonist. It should also be noted that both porcine and bovine Hb caused a slight inhibition of baseline SEAP activity when compared to the non-treated control cells. Hypothetically, these data may reveal a species-dependent restriction in Hb and TLR4 interactions with this ligand, and could potentially represent an evolutionarily-conserved mechanism in DAMP recognition. Indeed, a therapeutic RBC substitute, Sanguinate^TM^ (PEGylated carboxyhemoglobin bovine), is currently being examined in clinical trials [41,42,43], wherein its use avoids pathologies that typically include inflammation.

### 3.2. Assessment of NF-kB Activity in the Presence of Bvr

**Figure 5:**
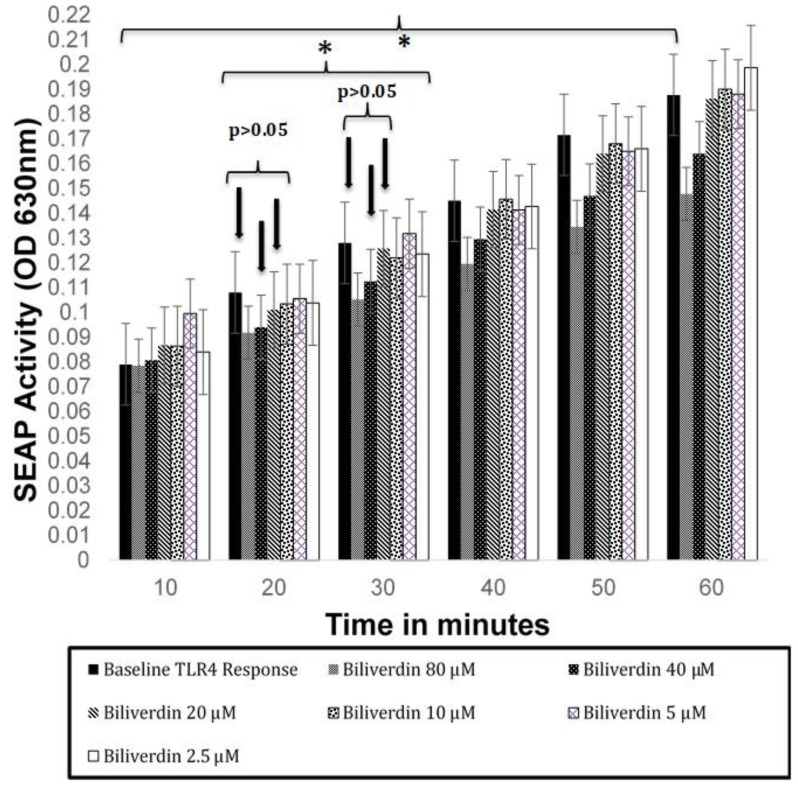
SEAP production by HEK-Blue TLR4 cells treated with Bv overnight. The following day, was measured at 630 nm. Because Bv is a poor TLR4 stimulant, the treatments were assayed at 10 min time intervals to assess stimulation or inhibition of innate NF-κB activation, as measured by dependent SEAP activity. This also demonstrated that Bv concentration has no direct effect on the measurement of SEAP activity. Otherwise, the cells used in these experiments were not stimulated with other TLR4 ligands and SEAP is measured as a baseline activity. Comparison of 10 min SEAP activity (QuantiBlue^TM^ conversion; OD 630 nm) to 60 min SEAP activity demonstrated that the baseline enzyme increased at a regular rate, regardless of Bv concentration. The increase in SEAP over the entire 60 min, and between the 20–30 min time intervals, were both found to be significant by ANOVA (*p* < 0.05), as shown by brackets and an asterisks (*). There were no significant differences (ANOVA) between media controls and the 20 or 40 μM Bv treated reporter cells at either the 20 or 30 min intervals (designated by arrows; *p* > 0.05).

Cultured HEK-Blue hTLR4 cells were treated with 100 μL of Bv diluted in complete media and tested in quadruplicate groups of wells. Before the inhibitory activity of Bv was assessed in ligand-stimulated cells, Bv was tested for inherent stimulatory and inhibitory activity in otherwise non-stimulated HEK-Blue hTLR4 cells (Figure 5). Dilutions from a stock solution of 100 μM Bv were made, based upon other studies wherein 10 μM Bv had been used [23]. The cells were incubated with or without Bv overnight. The day following stimulation, 20 ul of cell culture supernatant was removed from the treated cultures, added to the SEAP substrate, incubated at 37 °C with ambient CO_2_, and the OD 630 nm was assayed every 10 min for a total of 60 min. As noted above, SEAP activity in the culture supernatant was considered to be dependent upon NF-κB activity.

Inherent NF-κB activation that occurs in these cells without ligand stimulation was considered as the “baseline” activation. The kinetic presentation of baseline SEAP data was necessary here to conclude that increased SEAP activity was not an artifact of Bv being present in the final substrate reaction mixture, and that Bv did not reduce inherent NF-κB activity. In this respect, Bv treatment did not significantly increase the baseline NF-κB activity among otherwise non-stimulated cells. Furthermore, the data shows that SEAP activity steadily increased over the 60 min time course in all treatment groups, whether the cells were treated with Bv or not treated with Bv. It should be noted that 80 µM Bv reduced inherent SEAP production by 24% and 22% at only the 50 and 60 min time points (*p* < 0.05), respectively. Moreover, in our reporter cell model, Bv was not found to be a stimulatory TLR4 ligand. Further interpretation of the consistency in the kinetic data provides some evidence that Bv has little of no significant effect on the actual SEAP-reaction when concentrations below 80 µM Bv are used. Therefore, Bv concentrations from 20–40 μM were chosen for further evaluations of Bv inhibition. Furthermore, this concentration range was in the middle of the dilution curve, where NF-κB-dependent SEAP activity was minimal and approximated the non-treated cultures.

### 3.3. Bvr Inhibition of LPS-Induced TLR4 Activity

**Figure 6:**
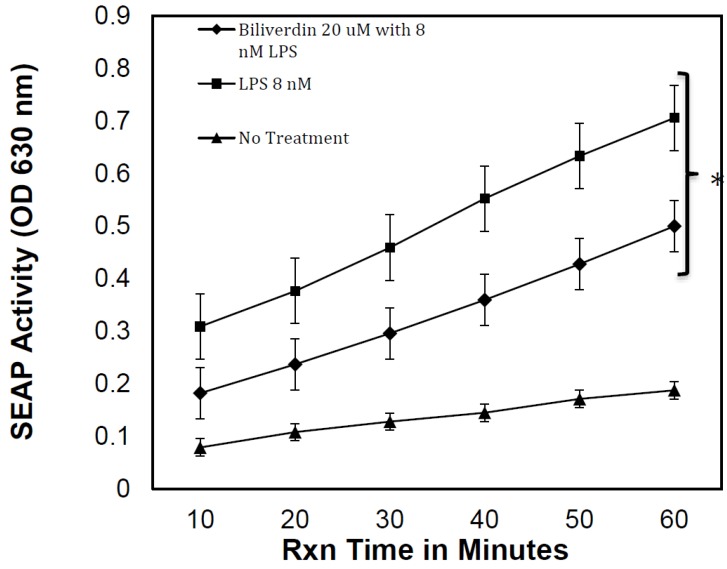
Kinetic evaluation of SEAP activity in supernatants derived from LPS-treated HEK-Blue hTLR4 cells. Cells at an initial density of 1 x 10^5^/well were cultured overnight with 8 ηM LPS (squares), 8 ηM LPS plus 20 uM Bv (diamonds), or media alone (triangles). Since it had been shown in Figure 5 that Bv alone was non-stimulatory and Bv inhibition of baseline was insignificant, Bv alone treatment was not reiterated here. The 8 ηM concentration of LPS used here corresponds to 0.8 µg/mL based upon a molecular weight of 100,000 (Sigma). A 60 min SEAP time course of substrate conversion was evaluated to show SEAP velocities over time (Slope LPS + Bv = 0.0065, R^2^ = 0.997; Slope LPS = 0.0085, R^2^ = 0.999; No Treatment Slope = 0.0021, R^2^ = 0.989). ANOVA measurement of significance between LPS and LPS + Bv is designated by an asterisk (*; where *p* < 0.05).

It has been reported that Bv and Bu improves the clinical outcome in experimental sepsis [24,44]. Herein, we tested Bv’s ability to modulate LPS activation in cultures of HEK-Blue hTLR4 cells. Based upon our prior experience, 4 µg/mL LPS will provide a more than adequate TLR4 activation of these cells, resulting in consistently reproducible SEAP production. Overnight stimulation was performed with 8 ηM (4 µg/mL) LPS, with or without 20 μM Bv. Time course enzymatic data were collected on culture supernatants to demonstrate that Bv did not directly alter SEAP enzymatic activity; OD 630 was measured every 10 min for a total of 60 min. Both the non-treated and LPS-treated cells produced kinetic curves with similar slopes, indicating that SEAP activity in the supernatants progressed at relatively similar rates over the 60 min test period. The SEAP kinetic curves derived from cells treated with media only, 8 ηM LPS only, and 8 ηM LPS plus 20 µM Bv are presented in Figure 6. Baseline SEAP activity was noted and increased two-fold during the 60 min incubation period. As expected, LPS stimulation increased baseline SEAP activity by 95%. The data show that 20 µM Bv significantly reduced SEAP activity at each of the 10 min time intervals (*p* < 0.05). When culture supernatants from LPS + Bv-treated cells are compared to LPS-only treated cells, the data show there was, on average, 35% inhibition in NF-κB-dependent SEAP activity. A minimum of 20 min of SEAP activity was needed to consistently observe Bv-induced inhibition. The slopes of the 60 min linear regressions were compared to verify inhibition. Using this approach, it was noted that Bv caused a 25% reduction in the SEAP reaction velocity (Slope LPS + Bv = 0.0065; Slope LPS = 0.0085).

### 3.4. Bvr Inhibition of FM-Induced TLR4

**Figure 7:**
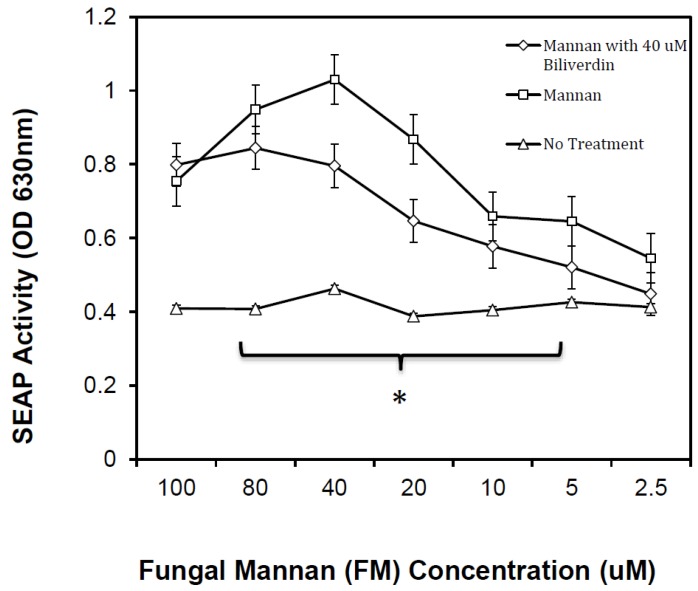
SEAP activity in culture supernatants derived from fungal mannan (FM)-treated HEK-Blue hTLR4 cells. Cells were plated at a cell density of 1 x 10^5^ cells/well and incubated overnight with dilutions of FM (Squares), dilutions of FM plus 40 μM Bv (Diamonds), or culture medium alone (Triangles). All FM and Bv dilutions were made in complete medium with FCS. The concentration range for FM is 2.5–100 μM and its calculation is based upon its polyacrylamide gel electrophoresis (PAGE) mobility of approximately 250 kD [29]. Changing from a kinetic evaluation/presentation to a concentration dependency was necessary in this series of experiments, since high concentrations of FM in excess of 160 μM are non-stimulatory for TLR4. FM was prepared from *C. albicans* as described in the Materials and Methods section. Linear regression analysis showed: No treatment, slope < 0, R^2^ = 0.00025; FM treatment, slope = 0.0033, R^2^ = 0.776; FM + Bv treatment, slope = 0.0022, R^2^ = 0.250. FM concentrations used in the range identified by the bracket were tested by an ANOVA, wherein FM treatment compared to FM + Bv treatment was found to be significantly different over the range designated by the bracket (*p* < 0.05 shown as *).

FM is the predominant TLR4 ligand found on the surface of many fungi. Its interactions with TLR4 produce reproducible activation via NF-κB that ultimately cause transcription of a set of inflammatory-related genes analogous to those activated with LPS. Ligand concentrations needed for FM activation can vary between FM preparations, as the targeted *O*-linked mannosylation epitope is pH labile. The FM used here was derived from *C. albicans* 20A using the cetavalon extraction method, and the inherent TLR4 ligand reactions are independent of LPS (as determined by polymyxin B depletion [29]). As noted in Figure 7, TLR4 reactivity to FM treatment was tested using a range of FM concentrations. A serial dilution of FM was necessary, because FM treatments using concentrations greater than 160 µM are not stimulatory in these cells, Viz. peak stimulation is achieved at 40 μM FM. Quadruplicates of HEK-Blue TLR4 cells were treated with FM or FM plus 40 µM Bv. The control untreated HEK-Blue TLR4 cells received complete media only. SEAP activity was measured at multiple time points, reported herein are the 30 min substrate conversions. The data show that 40 μM FM provided maximal HEK-Blue hTLR4 activation, wherein a 175% increase above baseline SEAP production was seen. The FM dilutions (2.5–100 μM) are shown in Figure 7 to demonstrate the inhibitory effect across the ligand dose range. The data show that FM stimulation was inhibited by an average of 25% when 40 µM Bv was used with 20-40 µM FM. Inhibition was not found to be significant when FM concentrations above 40 μM were used. A predicted molar ratio of 1:4–1:2 (Bv/FM) yielded the most pronounced inhibition.

### 3.5. Inhibition of Human Hb Activity on TLR4 by Bvr

**Figure 8:**
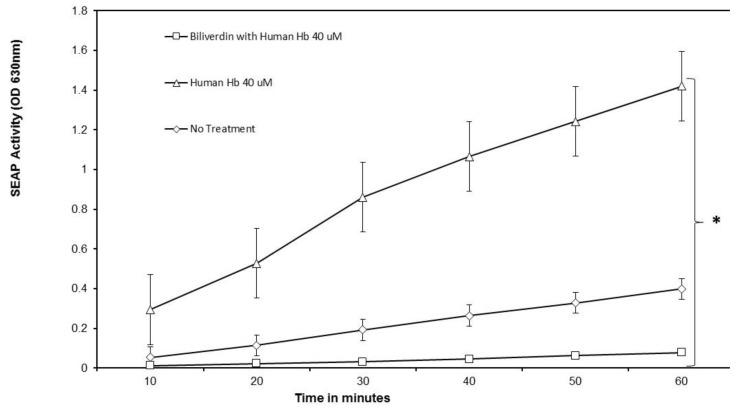
Kinetic evaluation of SEAP activity in culture supernatants derived from Hb-treated HEK hTLR4 cells. Cells were cultured at an initial density of 1 × 10^5^ cells/well and treated with 40 μM Hb (Triangles), 40 μM Hb plus 40 μM Bv (squares), or complete medium with FCS (diamonds). All Bv and Hb dilutions were made in complete medium plus FCS. SEAP was tested using QuantiBlue substrate (InvivoGen). The data are presented in kinetic format, wherein OD 630 nm was measured in reaction wells every 10 min. A kinetic analysis was performed to show any changes that might occur in both total SEAP activity and enzymatic rates (Hb slope = 0.040, R^2^ = 0.98; Hb + Bv slope = 0.007, R^2^ = 0.99; No Treatment slope = 0.0016, R^2^ = 0.98). Bv treatment only was shown in Figure 5 to have little or no effect on baseline SEAP and is not reiterated here. Significance between lines is shown with a bracket and an asterisk (*) designating *p* < 0.05 and was based upon the ANOVA results between the regression lines for Hb treatment only and Hb + Bv treatment.

The kinetic analysis of SEAP activity in culture supernatants derived from Hb-treated HEK-Blue hTLR4 cells is shown in Figure 8. As noted in previous experiments, Hb (40 μM final concentration) was added to 1 × 10^5^ HEK-Blue hTLR4 cells/well in 96–well plates with or without Bv (40 μM final concentration), and the cells were incubated overnight. Human Hb induced SEAP activities in all tested concentrations above 10 μM. A 1:1 ratio of 40 µM Bv and 40 μM human Hb mixture was tested with HEK-Blue TLR4 cells in quadruplicate wells. Untreated HEK-Blue TLR4 cells and 40 µM human Hb-treated HEK-Blue TLR4 served as controls. SEAP activities produced by HEK-Blue TLR4 that were treated with human Hb followed a linear pattern in relation to time. Hb treatments combined with Bv-treatments yielded diminished SEAP activities when compared to Hb alone treatments. This effect exceeded the inhibition seen when LPS or FM ligands were used. Repeated tests produced equivalent results, where Bv treatments reduced Hb-induced SEAP activity by an average of 95% (*p* < 0.05). As a result of Bv inhibition, the linear regressions depicting the Hb and Hb + Bv SEAP activity plots were quite dissimilar, with slope values of 0.040 versus 0.007, respectively.

## 4. Discussion

In mammals, the vast majority of Hb is compartmentalized in RBCs where its chief function is O_2_ and CO_2_ transport. In the less evolved forms of life, one of Hb’s primary roles is to function as a NO dioxygenase [25,37]. Once liberated from the RBC by lysis, Hb can freely react with NO, and its impact on the vasoactive role of NO in inflammation is amplified. Given the possible negative impact of Hb on NO homeostasis, mechanisms must exist that alert the innate immune system to control the effects of RBC lysis. Herein the role of TLR4 in damage recognition is linked to Hb, and the subsequent signaling cascade is paramount in limiting local tissue damage. Hb activation of NF-κB occurs via TLR4 stimulation and promotes local immune cells to initiate a cytokine cascade that directs localized inflammation towards healing. Hemolysis and cell free-Hb are dealt with rather swiftly by an elaborate scheme of scavenging and catabolic reactions. The consequences of Hb interacting with TLR4 is another potential anomaly, impacting cancer cell adaptation and survival. The chronic nature of cancer induced hemorrhaging can provide a constant source of Hb that both cancer cells and surrounding tissues could use to promote healing, but another set of consequences—including cancer cell survival and down regulation of cellular immunity—may also occur.

In the current study, we used an engineered human cell line, HEK-Blue hTLR4, to demonstrate the ability of TLR4 to recognize Hb [45]. These human cells co-express TLR4, CD14, and MD-2 on their membranes. TLR4 activation and the related signal cascade are both assessed by measuring the accumulation of SEAP in culture supernatant after overnight incubation with potential ligands. SEAP is produced only after NF-κB activation of a reporter plasmid. We initiated our study by comparing Hb with LPS as a TLR4 agonist. The results shown in Figure 2 confirmed that Hb was a capable TLR4 agonist, but was not nearly as effective as LPS. This data clearly demonstrated a convergence of innate anti-microbial functions with the innate response to injury. Redundancy in these responses most likely reflects both the need to debride injured tissue and the need to import immune cells for debris clearance. We did not evaluate cytokine production in these experiments—nonetheless, other published studies from our lab have shown that TLR4 signaling by LPS and FM induce TNF, IL-1, and COX-2 gene transcription in the HEK-Blue hTLR4 cell line [29]. NF-κB signaling in the engineered HEK-Blue hTLR4 reporter cells is a commonly used, well-characterized signaling model and is representative of the ligand reactions displayed by multiple cell types [29,30,31,33,46]. In light of these previous studies, we suggest that in vivo activation of TLR4 likely yields the same collection of cytokines, and these signals should influence local vascular endothelium by eliciting an inflammatory response that attracts phagocytic cells to the location of extravascular hemolysis. Once there, these immigrating cells are likely activated by additional RBC debris and more Hb. If this occurs in the tissue associated with a nest of cancer cells or a tumor, the infiltrating cells may further contribute to, or interfere with, ongoing anti-cancer immune responses.

As with any immune function, the quantity of ligand will dictate the strength and extent of the response. In our Hb dilution scheme, 150 μg/mL Hb was needed to achieve SEAP activity levels equivalent to those observed with 4–16 μg/mL LPS. Although this is a simplified scheme, a comparison of the SEAP produced by our ligand dilutions predicts that Hb activation of TLR4 requires roughly 10-fold more ligand than LPS. Given this estimate, we can extrapolate upon the generally accepted value of 160 mg of Hb/mL blood, by predicting that complete lysis of 1 mL of blood might produce the equivalent TLR4 activity as would 10 mg of LPS. In vivo hemoglobin is rapidly bound and neutralized by haptoglobin. With respect to injuries in tissue compartments where perfusion by plasma is limited, the eventful influx of acute phase proteins (including haptoglobin) may not replenish saturated haptoglobin and, as a result, cell-free Hb and its catabolites may accumulate.

To verify that the induced SEAP activity in these cells was dependent upon Hb’s interaction with TLR4, we hypothesized that Hb could contain LPS. To address the first concern, we utilized polymyxin spin columns (Sigma) to remove potential LPS contaminants [47]. The LPS-depleted Hb was no less effective as a TLR4 agonist than non-depleted Hb (Figure 1). In our hands, the HEK-Blue hTLR4 cells did not accurately detect LPS below 10 ng/mL. This suggests that LPS would have to be present in Hb preparations as a contaminant, representing 0.01% of the total Hb mass or 10 ng LPS/1 µg Hb. Since the polymyxin B column depletes 99.98% of the LPS in samples, this step adequately eliminates the feasibility of LPS contamination causing TLR4 activation. To address the possibility of a non-TLR4 related mechanism, we used the parental HEK-Blue Null cells as controls (Figure 2). These cells carry NF-κB-dependent SEAP genes on a reporter plasmid, but the cells do not respond to TLR4 ligands. They produce SEAP when stimulated with TNFα. It has been proposed by others that hemoglobin may serve, in its natural role, as an oxidizer and activate cells via another pathway. That was not observed here, as the Null cells remained non-responsive to both LPS and Hb.

We then compared Hb from three different mammals (Figure 3; human-, cow-, and pig-derived Hb) for their ability to stimulate TLR4. Human Hb is composed of two α chains and two β chains, and in adults this constitutes approximately 97% of the total Hb. Structurally speaking, cow Hb is quite similar to human Hb, but has functional differences in O_2_ binding that are due to amino acid-dependent structural differences in the A and E helices [48]. Porcine Hb shares about 85% sequence identity with human Hb, but also displays a reduction in oxygen-linked chloride binding [49]. The amino acid homology and oxygen binding differences in these xenogeneic Hb have made them popular candidates for building therapeutic blood substitutes. Bovine Hb has emerged as one of the more applicable acellular hemoglobin (Hb)-based oxygen carriers (HBOCs). With regards to the limited TLR4 comparisons made here, we demonstrated that Hb from pigs and cows was unable to stimulate NF-κB signaling among HEK-Blue hTLR4 reporter cells. Because we observed SEAP values lower than baseline, we also tested the viability of Hb-treated cells using a formazan dye assay (2,3-bis-(2-methoxy-4-nitro-5-sulfophenyl)-2H-tetrazolium-5-carboxanilide; XTT) following overnight stimulation with each of the three Hbs. Viability of Bv-treated cells was found to be equivalent or greater than non-treated controls. Hb-induced oxidation of XTT might have impacted this assay, but cells treated with human Hb displayed more XTT conversion than equivalent dilutions of bovine and porcine. XTT changes paralleled SEAP activity changes in cells treated with human Hb and were roughly equivalent in corresponding porcine- and bovine-Hb treatments, which failed to stimulate SEAP production. From these studies, it would appear that porcine and bovine Hb are far less stimulatory of hTLR4 than human Hb, and this feature—plus their distinct O_2_ carriage—may partly explain the successful clinical development as HBOCs.

The overall aim of this study was to show that Bv would differentially inhibit both microbial ligand and Hb activation through TLR4. To arrive at that goal, we had to first establish that Bv was not a stimulatory TLR4 ligand. Bv was tested in the cellular model at multiple concentrations, and a kinetic evaluation of SEAP was performed among the culture supernatants. If Bv is an inhibitor of NF-κB, the general effect of Bv should be observable with baseline SEAP activity. Baseline NF-κB activity represents the non-TLR4-stimulated, but normal NF-κB activity incurs as cells adhere to plastic or undergo stress. Since NF-κB can be activated during these events, the NF-κB-dependent SEAP reporter responds to NF-κB and produces baseline SEAP activity. Over the 60 min assay, we noted that baseline SEAP activity was detectable and significant (*p* < 0.05). Bv-inhibition of baseline was only observed with 80 μM Bv, and was only significant when substrate conversion was allowed to progress 50–60 min. Others have noted that treatment concentrations of ≤50 μM Bv displayed no toxicity in Raw 264.7 cells [23], which we also confirmed among Bv treatments of HEK-Blue TLR4 using an XTT viability/cytotoxicity assay. In this respect, we limited our use of Bv in future experiments to 20–40 μM concentrations and based our conclusions on reaction times of 20–40 min.

LPS and FM were chosen for comparison with Hb as targets for Bv-inhibition to determine if TLR4 recognition would remain intact, just as it might when Bv decelerates TLR4-dependent injury responses in vivo, and a generalized inhibition of NF-κB is one possibility. If this occurred, we would expect Bv to inhibit all three activation processes in equivalent fashion. A second possibility is that Bv would inhibit Hb’s activity, but not that of LPS and FM. The third possibility is that Bv would differentially inhibit Hb, LPS, and FM activation through TLR4. The latter mechanism would be advantageous in injured or infarcted tissue that becomes infected, and could be important with infectious microbes that cause hemolysis. We found evidence that the latter of the three possible activation/inhibition patterns occurred in this reporter cell model.

In the HEK-Blue hTLR4 cell model, the optimal Bv concentration range needed to alter LPS and FM reactivity in NF-κB signaling was 20–40 μM. Since LPS is a superior TLR4 ligand, we chose 8 ηM (0.8-1 μg/mL) LPS as an optimal dose. This amount of LPS routinely produces reliable SEAP activity that, although not equivalent, otherwise falls within the range of SEAP activity produced by Hb. Using FM as a ligand was more challenging, because FM is inferior to LPS as a TLR4 ligand, but FM’s ability to stimulate decreased when FM concentrations exceeded the optimal doses at 20-40 μM. This action by FM could be due to its nature as a multivalent mannosylated protein that can saturate TLR4 and its co-receptors. If Bv was competing for TLR4 or its receptors, we might expect FM to be more resistant to Bv inhibition than LPS. When Bv was applied to HEK-Blue hTLR4 cells with FM, the observed inhibition paralleled the activation curve. Inhibition was greatest at the optimal FM concentrations (20-40 μM), and no significant Bv inhibition was noted at the highest two FM concentrations. This relationship may be interpreted as either a competitive ligand relationship, or as a regulatory mechanism that resists NF-κB activation above a certain threshold. A possible mechanism that has been proposed by others [23] is that Bv down regulates TLR4 expression. Given that TLR4 is expressed in HEK-Blue hTLR4 reporter cells through transcription of a plasmid gene, such a mechanism would likely be targeting NF-κB regulation or its interaction with other transcription factors at the promoter site. Furthermore, this transcriptional macrophage model [23] was notably eNOS-dependent, but our study was performed in cells derived from HEK293 cells which have been reported to be eNOS negative [40,50,51].

To test Bv as an inhibitor of Hb-induced SEAP activity, we turned our focus to the kinetic approach. Hb’s activity as a ligand has been established in previous experiments (Figure 2), wherein the active concentrations had been identified. Moreover, a kinetic analysis was again employed to validate the SEAP response, and to identify changes that might be related to SEAP modification and an associated loss in activity. The slopes of the two reaction curves varied significantly (0.04 SEAP AU/min versus 0.007 SEAP AU/min). This may be due to Bv inhibiting SEAP activity in the reaction buffer; however, Bv did not display this characteristic when examined alone, wherein baseline SEAP activities increased substrate conversion at a steady rate (Figure 4). Moreover, Bv’s inhibition of Hb was more complete, insomuch as the data reflects a 95% level of inhibition. The data suggest that Hb-recognition by TLR4 could be compromised by the addition of Bv. Nevertheless, the signaling cascade was not completely abolished with LPS, and neither did it appear that SEAP activity was directly affected by Bv (slopes of LPS plus Bv = 0.065 SEAP AU/mL and of Bv alone = 0.085 SEAP AU/mL).

Collectively, these data lead us to believe that Bv could be a selective TLR4 antagonist, insomuch as the exogenous microbial ligands LPS and FM can still party stimulate TLR4 responses in the presence of Bv. We propose that Bv could interact within the receptor platform space that Hb would normally bind. Moreover, the nature of Bv antagonism was not established here, but the antagonistic variability noted between Hb and microbial ligands suggest that the antimicrobial reactivity of TLR4 is at least partially preserved in the presence of Bv.

We may expect that both Hb and Bv levels rise in local tissues during and/or after the development of various cancerous lesions. This and other studies show that Hb derived from hemolysis in lesions where the vasculature is compromised may alter cytokine production. A clear and present target for Hb-induced immune modulation is TLR4. A likely mechanism for local cytokine transcription is shown here as NF-κB signaling. Studies have shown Hb can cause oxidative stress, contributing to the overall systemic inflammatory response [52]. During this inflammatory response, heme and Hb can exceed scavenging systems, remain elevated, and enhance gene expression of inflammatory markers. Other studies [51] demonstrate administration of Bv during medical procedures, such as angioplasty, can protect against such responses by reducing intimal hyperplasia, thereby serving as therapeutic adjuncts. In the context of cancer, this role of Hb could support metastatic growth through the activation of NF-κB in TLR4 positive cancer cells, or through the activation of local tissue cells wherein effective anti-cancer immune functions are sacrificed in lieu of tissue inflammation and healing. In either case, Bv may also contribute by limiting the Hb response and allowing for continued anti-cancer responses.
